# Topographic Anatomy and Clinical Impact of Vascular Foramen on the Trochlear Groove of Adult Human Dry Femora

**DOI:** 10.7759/cureus.19732

**Published:** 2021-11-18

**Authors:** Muhammad Haris, Najma Baseer, Sobia Haris, Noman Ullah Wazir, Farah Deeba

**Affiliations:** 1 Department of Anatomy, Nowshera Medical College, Nowshera, PAK; 2 Department of Anatomy, Institute of Basic Medical Sciences, Khyber Medical University, Peshawar, PAK; 3 Department of Medical Education, Nowshera Medical College, Nowshera, PAK; 4 Department of Anatomy, Peshawar Medical College, Peshawar, PAK

**Keywords:** vascular foramen, trochlear groove, topography, human, dry femora, clinical impact, anatomy, adult

## Abstract

Objective

In this study, we aimed to assess the topographic anatomy and clinical impact of the vascular foramen on the trochlear groove of adult human dry femora.

Materials and methods

The incidence, shape, size, and location of the foramen present on the trochlear groove of the distal femur were studied using 33 adult human dry femora of unknown age and gender at the Department of Anatomy at Nowshera Medical College, Nowshera, Pakistan. Using a divider and scale, the incidence and structure of the vascular foramen in the trochlear groove were examined and its position in relation to the trochlear groove's upper and lower articulating edges was recorded.

Results

Out of 33 femora, 20 (60.6%) were right-sided and 13 (39.4%) were left-sided. All the foramina had round morphometry. In each of the 33 adult human dry femora, 15 (45.5%) had a single trochlear vascular foramen. One solitary trochlear vascular foramen was found in eight (53.3%) of the left femora and seven (46.7%) of the right femora. The diameter of each trochlear vascular foramina was about 2.5-4.5 mm with a mean diameter of 3.5 mm. Trochlear groove upper margin and foramen were 1.75-2.5 cm apart, whereas the lower margin was 0.5-01 cm apart. On average, foramen was 2.2 cm away from the upper margin, and 0.8 cm away from the lower margin. In the midline, nine (60%) trochlear vascular foramina were found, with five (55.5%) resting on the trochlear groove lower margin, and four (44.5%) lying mid-way at varying places from the upper and lower margins. The left of the centerline had six (40%) of the trochlear vascular foramen, whereas no trochlear vascular foramen was seen on the right side of the midline.

Conclusions

Based on our findings, a significant segment of our population has trochlear vascular foramen present on the trochlear groove as the rate of occurrence of this was found to be 45.5%. The trochlear groove center has the most foramen, accounting for 60% of all the foramen.

## Introduction

Typical long bones have an extremely vascular structure, which is supplied by four artery systems: periosteal, metaphyseal, diaphyseal, and epiphyseal arteries, which are commonly known as nutrient arteries [1]. The femur bone, which is the only long bone of the thigh, is nourished by four perforating, lateral circumflex, and descending genicular arteries [1]. Such arteries enter through many vascular foramina termed nutrient foramina [1]. This foramen is the entry point for nutrient artery in the long bones, which then flows into the marrow cavity via the nutrient canal [1]. Surgeons should have adequate knowledge about the nutrient vascular foramen's exact and precise position in order to avoid intraoperative harm to the artery running through it [2]. Furthermore, knowing where vascular foramina are located is essential for separating them from the rupture line [3]. Various scholars have characterized vascular foramen on the lower, upper, and shaft femur. Moreover, surgeons should also have sufficient knowledge about the morphometry and morphology of these foramina, which is useful in certain operative procedures in orthopedics as well as in plastic and reconstructive surgery, to avoid damage to the nutrient arteries [4]. In this study, our goal was to assess the topographic anatomy and clinical impact of the vascular foramen on the trochlear groove of adult human dry femora.

## Materials and methods

Study design and setting

This was a descriptive cross-sectional study conducted at the Department of Anatomy at the Nowshera Medical College, Nowshera, Pakistan.

Study duration, sample size, and sampling technique

After obtaining ethical approval from the Institutional Ethical Review Board (IERB) of the Nowshera Medical College vide letter No. 25/NMC/IERB/Sec dated 15/02/2021, this study was conducted in September 2021. A total of 33 adult human dry femora of unknown age and gender were included and studied. Non-probability convenience sampling was employed in the analysis.

Sample selection

Only those femora with undamaged lower ends were examined in this study, whereas those that had injured lower ends were excluded.

Data collection

Data were collected and entered into a Microsoft Excel 2010 spreadsheet. The incidence, structure, size, and position of vascular foramen found on the patellar surface of the femur, commonly recognized as the trochlear groove, were examined. Because the foramina were found in a variety of places, they were divided into four groups to aid orthopedic surgeons undertaking surgery on the distal femur. The first group consisted of the trochlear groove to the left of the midline. The second group comprised those in the midline, varying in distance from the top and lower trochlear groove edges. Those on the trochlear groove bottom edge, in the midline, were placed in the third group. The fourth group was made up of those on the trochlear groove to the right of the midline. The structure and size were examined for each foramen, and a divider and scale were used to measure trochlear groove diameter and distance from the top as well as bottom edges.

Data analysis

Foramen sizes and distances from the top and bottom borders of the trochlear groove were measured, and the incidence, range, and mean were calculated using SPSS Statistics version 24 (IBM, Armonk, NY).

## Results

**Figure 1:**
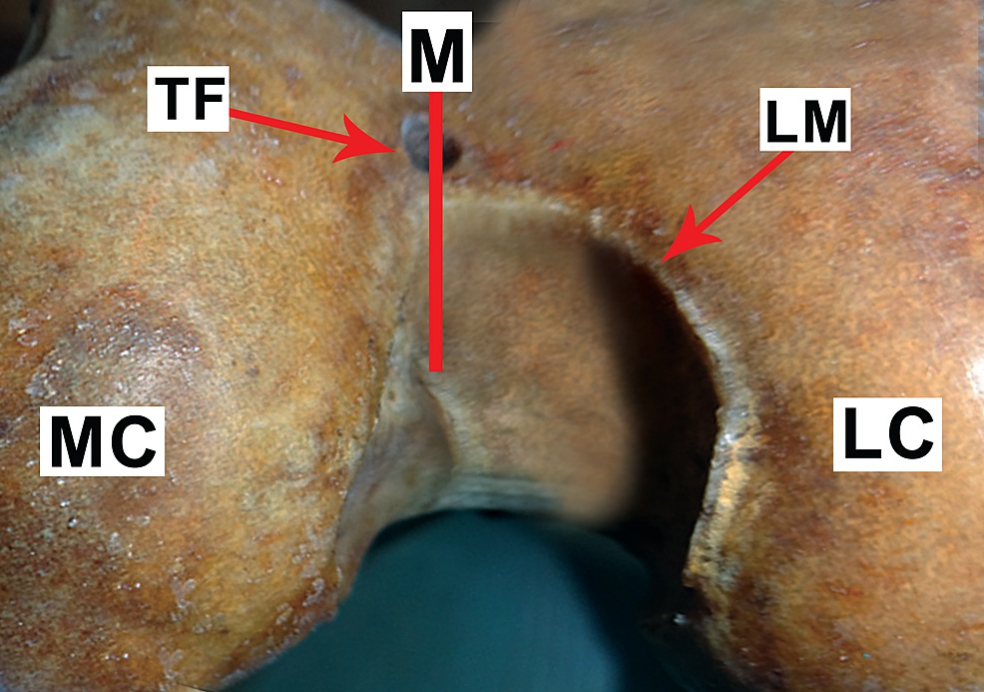
Vascular trochlear foramen in the midline at the lower margin of the trochlear groove TF: trochlear foramen; M: midline; LM: lower margin; LC: lateral condyle; MC: medial condyle

**Figure 2:**
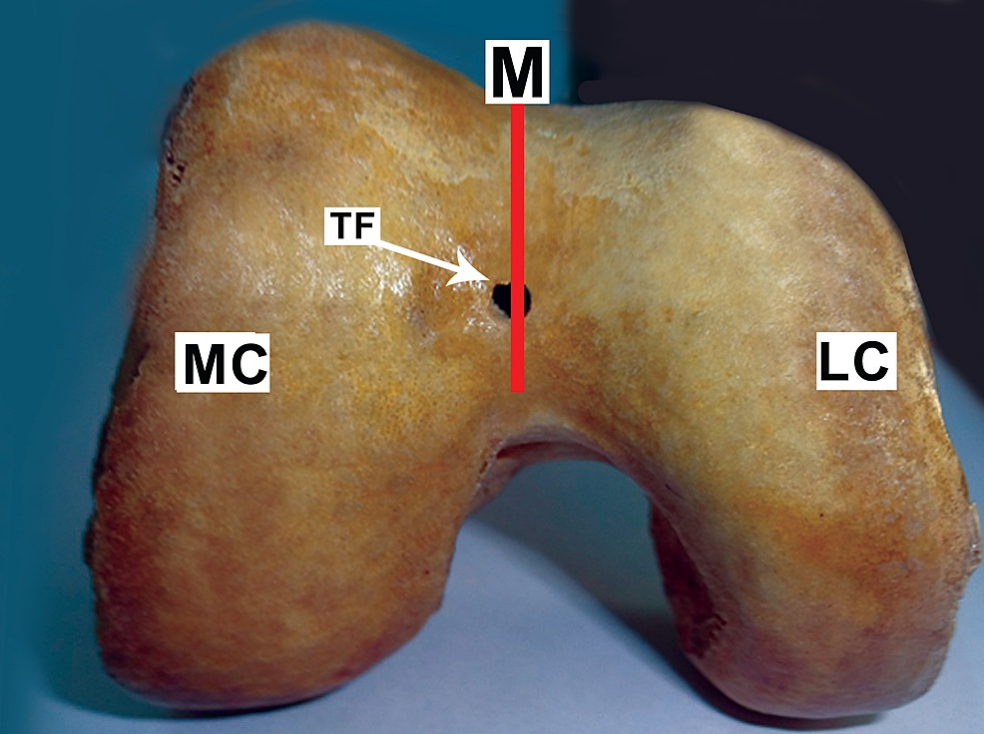
Vascular trochlear foramen in the midline on the trochlear groove of the distal femur TF: trochlear foramen; M: midline; LC: lateral condyle; MC: medial condyle

**Figure 3:**
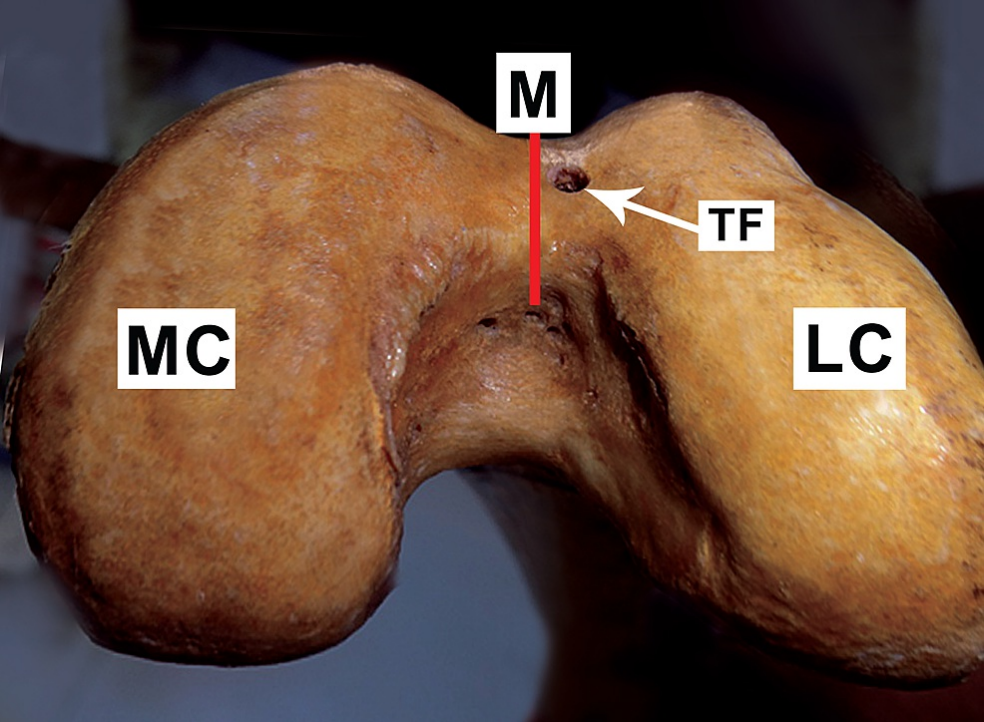
Vascular trochlear foramen on the left of the midline on the trochlear groove of the distal femur TF: trochlear foramen; M: midline; LC: lateral condyle; MC: medial condyle

Out of 33 femora, 20 (60.6%) were right-sided and 13 (39.4%) were left-sided. All the foramina had round morphometry. In each of the 33 adult human dry femora, 15 (45.5%) femora had a single trochlear vascular foramen. One solitary trochlear foramen was detected in eight (53.3%) of the left femora and seven (46.7%) of the right femora. The diameter of each trochlear vascular foramina was about 2.5-4.5 mm with a mean diameter of 3.5 mm. Trochlear groove upper margin and foramen were 1.75-2.5 cm apart, whereas the lower margin was 0.5-01 cm apart. On average, foramen was 2.2 cm away from the upper margin, and 0.8 cm away from the lower margin. In the midline, nine (60%) trochlear vascular foramina were found, with five (55.5%) resting on the trochlear groove lower margin as shown in Figure 1, and four (44.5%) lying in mid-way at varying places from the upper and lower margins as shown in Figure 2. The left of the centerline had six (40%) trochlear vascular foramen as shown in Figure 3, whereas no trochlear vascular foramen was seen on the right side of the midline.

## Discussion

Previous studies have extensively described vascular foramina in the supra, medial, lateral, and intercondylar fossa of the distal femur. However, despite a thorough literature search, we did not find a sufficient number of studies on the incidence, shape, size, and topographic anatomy of vascular foramen situated in the trochlear groove of the distal femur. In this study, 15 out of the 33 adult human dry femora of unknown age and gender had the trochlear vascular foramen, indicating a 45.5% occurrence rate.

According to Singh [5], trochlear vascular foramina were identified in 55.6% of the femora, which somewhat corresponds to our findings. The vascular foramen anatomical properties, such as its number, position, size, structure, and orientation, are crucial elements to consider in orthopedic procedures such as bone grafting and fracture repair. Because healing is dependent on blood flow, which is dependent on the aforementioned parameters, these factors are critical in determining the prognosis following a fracture [6]. The frequency of vascular foramina in a location is crucial because the more foramina there are, the more vascular networking there is. Having a significant number of foramina is advantageous since it ensures that the area is well-supplied, thereby enabling fast curing [7]. Providing adequate periosteal bloodstream to the bone permits significant corticocancellous bone fold harvest, which will abstain from endangering nutrition, in addition to the enormous trochlear foramen found in our study [8]. Because of the cambium layer's strong osteogenic potential and the bigger size of the vascular foramen, this location is optimal for avoiding bone non-union. A popular surgery for readjusting a varus or valgus leg posture is osteotomies of the distal femur. However, this operation frequently causes a significant amount of blood loss. As a result, during surgery, the periosteal feeders or bigger arteries that surround the bone wounds should be safeguarded [9].

The distal femur is important from a morphological, functional, and clinical perspective because of its location, structure, and weight-bearing role, which exposes it to substantial morbidity [10]. The joint of the knee and inferior part of the femur are normally implicated in osteoarthritis and fractures, and vascularized bone transplants are routinely performed on these areas [11,12]. The nutrition artery's blood flow is critical in free vascular bone grafting and should be retained to enhance rupture repair. The lower and upper ends of the femur and those of the tibia are the developing ends of the lower limb respectively [13]. The loss of vascular supply is one of the variables that cause late or non-union of ruptures. It is well-known that the healing of a traumatic or medically produced rupture takes a long time. As a result, orthopedic surgeons doing an open reduction of a fracture must have a morphological awareness of the vascular foramen in order to avoid harming the nutritive artery and minimizing the likelihood of non-union or malunion of the fracture [13]. The vascular foramen's exterior entrance is placed at a specific location in each bone. Vascular foramen was discovered in three distinct sites in our case: in the midline, there were 16 maximum foramina, two were present in the centerline of the inferior articulating boundary of the patellar surface, and seven were present to the left side. There is scarce research to compare the foramen's position. According to mythology, the vascular foramen was originally located near the ossification center because the arteries that inhabit it are descendants of those that first entered the ossifying cartilage [14].

According to Su et al. and Pereira et al., the developing bone may drag and burst the artery; the nutrition artery goes far from the growing end. As a result, vascular foramina are relocated away from the plant's growing edge. Two variables influence the positioning of vascular foramina: rates of growth at both bones and shaft end remodeling. The rates of bone growth and remodeling in persons of various ages should be investigated. Furthermore, fetal bones may be useful in deciphering this idea. These parameters could not be examined in the current investigation since it involved adult dry bones. The majority of trochlear foramina (64%) were found in the mid-way of the trochlear groove, whereas 28% were found on the left condyle, and 8% were detected along the bottom margin of the trochlear groove, somewhat to the left of the midline. Since there is no previous work in the literature that details the position of the trochlear foramen, there was no way to make a comparison [15,16].

The irregular orientation of vascular foramina might be caused by the stress of muscle attachments on the periosteum. The foramen can be a source of weakness in certain people, and it can fracture when strained, whether through greater physical activity or deterioration of bone quality. The position of the rupture in relation to the vascular foramen of elongated bone and the morphologies of edema are secondary symptoms in the diagnosis of this kind of fracture. Hence, orthopedic surgeons must be aware of the morphometry, morphology, and topographic anatomy of the trochlear vascular foramen in the trochlear groove of the distal femur.

## Conclusions

Our findings concluded that a significant proportion of our population has trochlear vascular foramen present on the trochlear groove since the occurrence of this was found to be 45.5%. The trochlear groove center has the most foramen, accounting for 60% of all the foramen. The morphologic, morphometrical, and topographical understanding of this foramen is indispensable for orthopedic and trauma surgeons. Sufficient knowledge is mandatory for the purpose of bone grafting, tumor resections, traumas, congenital skeletal deformities, and equipment and fixation, as well as graft techniques utilized in orthopedics and other procedural processes in the distal femur and knee region to maintain the normal blood flow. Furthermore, knowing where this foramen is located is critical for bone ossification, bone curing, and microvascular bone transplantation.
